# Disease trajectories in interstitial lung diseases – data from the EXCITING-ILD registry

**DOI:** 10.1186/s12931-024-02731-3

**Published:** 2024-03-06

**Authors:** Katharina Buschulte, Hans-Joachim Kabitz, Lars Hagmeyer, Peter Hammerl, Albert Esselmann, Conrad Wiederhold, Dirk Skowasch, Christoph Stolpe, Marcus Joest, Stefan Veitshans, Marc Höffgen, Phillen Maqhuzu, Larissa Schwarzkopf, Andreas Hellmann, Michael Pfeifer, Jürgen Behr, Rainer Karpavicius, Andreas Günther, Markus Polke, Philipp Höger, Vivien Somogyi, Christoph Lederer, Philipp Markart, Michael Kreuter

**Affiliations:** 1 Center for Interstitial and Rare Lung Diseases, Thoraxklinik, University of Heidelberg, German Center for Lung Research (DZL), Heidelberg, Germany; 2 Medical Clinic II, Pneumology and Intensive Care Medicine, Klinikum Konstanz, Konstanz, Germany; 3 Clinic of Pneumology and Allergology, Center of Sleep Medicine and Respiratory Care, Hospital Bethanien Solingen, Solingen, Germany; 4 Chest Clinic Immenhausen, Immenhausen, Germany; 5 Outpatient center for pulmonology, Warendorf, Germany; 6 Outpatient center for pulmonology, Fulda, Germany; 7 Department of Medicine II, University Hospital Bonn, Bonn, Germany; 8 Outpatient center for pulmonology, Ibbenbüren, Germany; 9 Outpatient center for pulmonology and allergology, Bonn, Germany; 10 Outpatient center for pulmonology, Böblingen, Germany; 11 Outpatient center for pulmonology, Rheine, Germany; 12 Institute of Health Economics and Healthcare Management, Helmholtz Center Munich GmbH, German Research Center for Environmental Health, German Center for Lung Research (DZL), Comprehensive Pneumology Center Munich (CPCM), Neuherberg, Germany; 13 IFT Institut für Therapieforschung, Center for Mental Health and Addiction Research, Munich, Germany; 14 Outpatient center for pulmonology, Augsburg, Germany; 15 Medical Clinic II, University of Regensburg and Klinikum Donaustauf, Donaustauf, Germany; 16 Department of Medicine V, Comprehensive Pneumology Center, LMU University Hospital, LMU Munich, German Center for Lung Research (DZL), Munich, Germany; 17 Patient Support Group Lungenfibrose e.V., Essen, Germany; 18 Medical Clinic II, University Hospital Giessen, Universities of Giessen and Marburg Lung Center (UGMLC), German Center for Lung Research (DZL), Giessen, Germany; 19 Pulmonary and Critical Care Medicine, Agaplesion Evangelisches Krankenhaus Mittelhessen, Giessen, Germany; 20 Mainz Center for Pulmonary Medicine, Departments of Pneumology, ZfT, Mainz University Medical Center and of Pulmonary Critical Care & Sleep Medicine, Marienhaus Clinic Mainz, Mainz, Germany; 21 Medical Clinic V (Pneumology), Cardiothoracic Center, University Medicine Marburg, Campus Fulda, Fulda, Germany

**Keywords:** ILD, IPF, Progression, Mortality, Risk factors

## Abstract

**Background:**

Interstitial lung diseases (ILD) comprise a heterogeneous group of mainly chronic lung diseases with different disease trajectories. Progression (PF-ILD) occurs in up to 50% of patients and is associated with increased mortality.

**Methods:**

The EXCITING-ILD (Exploring Clinical and Epidemiological Characteristics of Interstitial Lung Diseases) registry was analysed for disease trajectories in different ILD. The course of disease was classified as significant (absolute forced vital capacity FVC decline > 10%) or moderate progression (FVC decline 5–10%), stable disease (FVC decline or increase < 5%) or improvement (FVC increase ≥ 5%) during time in registry. A second definition for PF-ILD included absolute decline in FVC % predicted ≥ 10% within 24 months *or* ≥ 1 respiratory-related hospitalisation. Risk factors for progression were determined by Cox proportional-hazard models and by logistic regression with forward selection. Kaplan-Meier curves were utilised to estimate survival time and time to progression.

**Results:**

Within the EXCITING-ILD registry 28.5% of the patients died (n = 171), mainly due to ILD (n = 71, 41.5%). Median survival time from date of diagnosis on was 15.5 years (range 0.1 to 34.4 years). From 601 included patients, progression was detected in 50.6% of the patients (n = 304) with shortest median time to progression in idiopathic NSIP (iNSIP; median 14.6 months) and idiopathic pulmonary fibrosis (IPF; median 18.9 months). Reasons for the determination as PF-ILD were mainly deterioration in lung function (PFT; 57.8%) and respiratory hospitalisations (40.6%). In multivariate analyses reduced baseline FVC together with age were significant predictors for progression (OR = 1.00, p < 0.001). Higher GAP indices were a significant risk factor for a shorter survival time (GAP stage III vs. I HR = 9.06, p < 0.001). A significant shorter survival time was found in IPF compared to sarcoidosis (HR = 0.04, p < 0.001), CTD-ILD (HR = 0.33, p < 0.001), and HP (HR = 0.30, p < 0.001). Patients with at least one reported ILD exacerbation as a reason for hospitalisation had a median survival time of 7.3 years (range 0.1 to 34.4 years) compared to 19.6 years (range 0.3 to 19.6 years) in patients without exacerbations (HR = 0.39, p < 0.001).

**Conclusion:**

Disease progression is common in all ILD and associated with increased mortality. Most important risk factors for progression are impaired baseline forced vital capacity and higher age, as well as acute exacerbations and respiratory hospitalisations for mortality. Early detection of progression remains challenging, further clinical criteria in addition to PFT might be helpful.

**Supplementary Information:**

The online version contains supplementary material available at 10.1186/s12931-024-02731-3.

## Background

Interstitial lung diseases (ILD) comprise more than 200 mainly chronic diseases affecting the lung parenchyma due to inflammation and/or fibrosis [1, 2]. The multidisciplinary diagnostic process considers clinical, radiological, and pathological aspects of the disease [2, 3]. A relevant number of both, inflammatory and fibrotic ILD patients shows a progressive course of disease (PF-ILD) characterised by worsening of respiratory symptoms and decline in lung functional parameters such as vital capacity (VC). Recently a definition of progressive pulmonary fibrosis (PPF) has been published considering radiological evidence of progression as well as worsening of respiratory symptoms and functional decline [4]. Progression, at least in fibrotic ILD (fILD), is associated with increased mortality [5, 6]. Close monitoring is therefore important to detect progression as early as possible [7]. This is also relevant with regard to new therapeutic options, e.g. antifibrotic therapy in PPF. In the past years, antifibrotic drugs have been investigated in PPF [8–10]. Within the INBUILD trial, nintedanib showed efficacy in attenuation of FVC decline [8]. The RELIEF study investigated the efficacy of pirfenidone in PPF, and although the trial was stopped early due to under-recruitment, it showed a significant slowing of disease progression [9]. Another study studied pirfenidone in unclassifiable ILD (uILD) with progressive fibrosis. Although the primary endpoint based on home spirometry was not met, secondary endpoints such as on-site FVC, diffusing capacity for carbon monoxide (DLCO) and 6-MWD (6 min walking distance) were suggestive of effect of pirfenidone treatment [10].

In order to ensure early detection of progression in different ILD subtypes with a high degree of diagnostic certainty, further characterisation and understanding of disease behaviour is essential. Registries allow important insights on such aspects. The Canadian Registry for Pulmonary Fibrosis (CARE-PF) enrolled patients with fILD of any subtype prospectively, identified associated baseline factors, clinical characteristics and outcomes [11]. Progression was common in this cohort, and similarly prevalent in idiopathic pulmonary fibrosis (IPF) and hypersensitivity pneumonitis (HP) [11]. In line with this, prevalence of PF-ILD was reported to be 27% of al non-IPF patients in the retrospective PROGRESS study [12]. An international survey estimated progression in 14–32% of patiens with ILD other than IPF [13].

Our analyses are based on the “Exploring Clinical and Epidemiological Characteristics of Interstitial Lung Diseases” (EXCITING-ILD) registry. This multicenter, noninterventional prospective and observational disease and outcomes registry was conducted by the German Center for Lung Research (DZL) in close collaboration with cross-sectional sites [14]. Aim of the current work was to assess the following three objectives: (a) assessing progression in different ILD subtypes including IPF and other form of fibrotic and non-fibrotic ILD, (b) assessing risk factors for ILD progression and (c) analysing association between ILD progression and mortality.

## Methods

### Study Design

Within the EXCITING-ILD registry, sociodemographic and medical data on all different ILD subtypes were collected. The study protocol has been published elsewhere [14]. To summarise, incident and prevalent ILD patients from various healthcare facilities including outpatient, inpatient, and academic sites, were included. All patients were followed prospectively for a minimum of 36 months and a maximum of five years. Data from baseline and follow-up visits were reported by the investigators and entered into the full analysis set (FAS): demographic data, information on ILD subtypes, diagnostic procedures, distinct comorbidities, ILD management, as well as outcomes, progression and associated factors. For further analyses all patients with a minimum of one documented post-baseline visit during a median follow-up of 3 years were considered [14, 15]. The study was approved by the Ethics Committee of the Medical Faculty of the University of Heidelberg, Germany (S-525/2013) as well as by all local ethics committees of the participating centers.

### Statistical analysis

Means with standard deviations (SD) and percentages were used to analyse observational data descriptively. For graphical presentation lineplots were generated for changes from baseline in FVC, and alluvial plots for transitions of progression stages. Kaplan-Meier curves were utilised to estimate progression-free survival times (survival time, time to progression), for the comparison of two or more Kaplan-Meier curves a log-rank test was performed. For these analyses, individuals who do not experience the event until the registry was closed, who were lost to follow up or withdraw from the registry are censored. Then the earlier available date of last follow-up or discontinuation was used. Confidence intervals were set with a two-sided level of 95% [14]. To quantify the difference between groups, hazard ratios complemented by corresponding two-sided 95% confidence intervals were estimated based on a Cox proportional hazards model. P-values of the corresponding Wald-test were calculated. For data analysis the statistics software R (R version 4.1.2) was used.

The following parameters were applied for the definition of ILD progression during time in registry: (1) Progression was classified on the basis of absolute changes in FVC % predicted as established by Hoffmann-Vold et al. [16] into significant (FVC decline > 10%) or moderate progression (FVC decline 5%–10%), stable (FVC decline or increase < 5%) and improvement (FVC increase ≥ 5%). The reference time was chosen as first visit with a non-missing FVC value. Patients were considered as progressive, if progression had occurred in at least one follow-up visit. (2) A second definition for PF-ILD was set as absolute decline in FVC % predicted ≥ 10% wthin a period of 24 months *or* at least one respiratory hospitalisation.

ILD-GAP index was calculated for each patient. The ILD-GAP index is a point scoring stage model based on clinical and physiologic variables to predict mortality in patients with different forms of ILD. Higher ILD-GAP scores indicate worse prognosis [17].

To develop a model to predict progression and to identify variables with a significant influence on progression, logistic regression with forward selection was used. As possible predictors the following variables at baseline were considered: time since baseline visit, age at year of inclusion, sex, body mass index (BMI), familial ILD, ILD subtype, smoking behaviour, FVC in % predicted, DLCO-SB in % predicted, reflux, pulmonary hypertension, concomitant emphysema. The same model was used to predict FVC changes. First, univariable regression models were set up (5% significance level) and second multiple regression models were generated.

Survival time was defined as the time between date of diagnosis and date of death. Another analysis was made for survival time from inclusion to the registry. Outcome analyses were made for a selection of ILD subtypes of special interest; these included IPF, non-specific interstitial pneumonia (iNSIP), cryptogenic organizing pneumonia (COP), uILD, sarcoidosis, HP, rheumatic and connective tissue diseases with pulmonary involvement (CTD-ILD), and drug-related ILD (DI-ILD).

## Results

### Study population

The EXCITING-ILD registry included 601 patients of 32 centers with a mean age of 64.3 years (Table 1). 60.7% were male and mean FVC was 76.4% predicted. The ILD subtypes included were: 26.6% sarcoidosis, 25.3% IPF, 9.7% HP, 7.2% CTD- and RA-ILD, 7% iNSIP, 5.7% uILD, 4.2% COP, 2.7% DI-ILD, 2.3% fibrosis in emphysema patients without signs of other ILD (CPFE), 1.8% pneumoconiosis, 1.2% pulmonary lymphangioleiomyomatosis (LAM), 1.0% eosinophilic pneumonia, 1.0% radiotherapy associated-ILD (RTX-ILD), 0.8% other granulomatous lung disease (other GRAN-ILD), 0.8% desquamative interstitial pneumonia (DIP), 0.7% respiratory bronchiolitis-associated interstitial lung disease (RB-ILD), 0.5% pulmonary alveolar proteinosis (PAP), 0.3% pulmonary Langerhans´ cell histiocytosis (PLCH), and 1.2% others.

**Table 1:** Baseline characteristics of the full analysis set

Baseline characteristics		Total (n = 601)
Male Sex, n (%)		365 (60.7)
Age [years], mean (SD)		64.3 (14.2)
Current smoker, n (%)		52 (8.7)
Ex-smoker, n (%)		270 (44.9)
FVC [% predicted], mean (SD)		76.4 (20.8)
FEV1 [% predicted], mean (SD)		79.2 (20.0)
DLCO-SB [% predicted], mean (SD)		54.1 (21.6)
ILD-GAP-Index, n (%)	I	275 (45.8)
	II	164 (27.3)
	III	128 (21.3)
	Missing	34 (5.7)
Time to diagnosis in months, mean (SD)		-38.8 (64.4)
Disease duration at diagnosis in months, mean (SD)		13.2 (27.4)

The reference point of time for time differences is the inclusion date, e.g. time to diagnosis was defined as time date of diagnosis to date of inclusion. SD = standard deviation, FVC = forced vital capacity, FEV1 = forced expiratory volume in 1 s, DLCO = diffusing capacity for carbon monoxide (CO)

### Disease trajectories

**Fig. 1:**
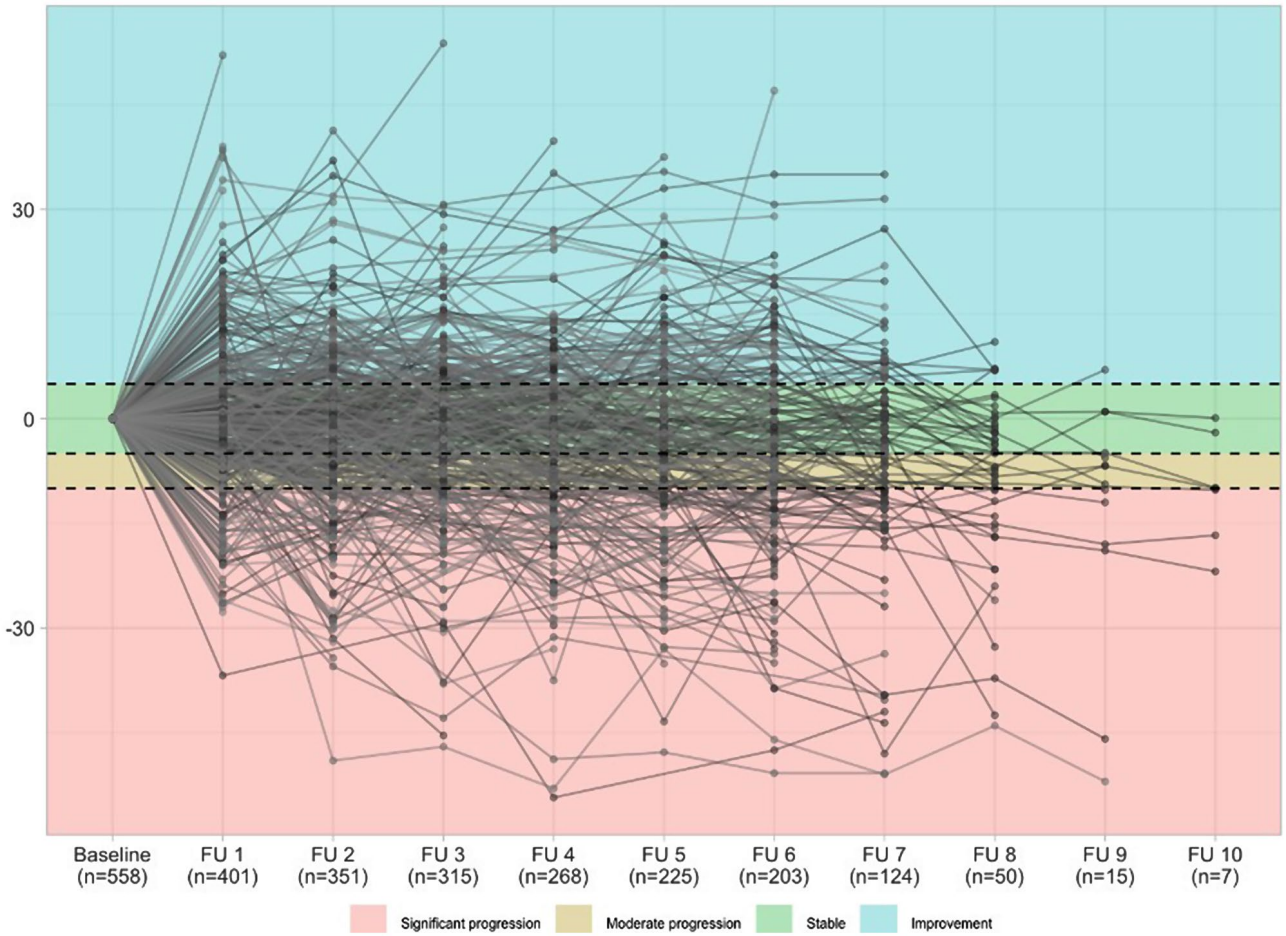
Lineplot with individual changes from baseline in FVC [% predicted]. Progression was classified as described above and is shown in four different colours. Only individuals with non-missing values are shown (n = 558). The frequencies are shown for each follow-up visit (FU) starting with the first follow-up visit (FU 1) after 6 months, FU 2 after 12 months, FU 3 after 18 months, etc. FVC = forced vital capacity

Progression based on absolute changes in FVC % predicted was detected in more than half of the patients (n = 304, 50.6%) with significant progression occurring more frequently than moderate progression, e.g. 15.5% vs. 10.3% for follow up visit 5, accordingly after 2.5 years in the registry. The rate of progressive patients increased over time while 32.4% of patients demonstrated stable FVC values or improvement *-* with heterogeneous individual disease trajectories (Table 2; Fig. 1).

**Table 2:** Frequency of progression stages grouped by visit

	Baseline (n = 601)	FU 1 (n = 561)	FU 2 (n = 493)	FU 3 (n = 435)	FU 4 (n = 395)	FU 5 (n = 354)	FU 6 (n = 321)	FU 7 (n = 207)	FU 8 (n = 75)	FU 9 (n = 16)	FU 10 (n = 8)
Significant progression	0	51 (8.7%)	64 (12.4%)	66 (14.4%)	65 (15.7%)	57 (15.5%)	53 (16.1%)	39 (17.4%)	15 (19.0%)	6 (35.3%)	3 (33.3%)
Moderate progression	0	56 (9.5%)	56 (10.9%)	44 (9.6%)	46 (11.1%)	38 (10.3%)	36 (10.9%)	16 (7.1%)	10 (12.7%)	4 (23.5%)	2 (22.2%)
Stable	561 (93.3%)	201 (34.2%)	145 (28.1%)	123 (26.9%)	103 (24.9%)	83 (22.6%)	63 (19.1%)	44 (19.6%)	20 (25.3%)	4 (23.5%)	2 (22.2%)
Improve-ment	0	103 (17.5%)	93 (18.0%)	91 (19.9%)	61 (14.8%)	52 (14.1%)	57 (17.3%)	30 (13.4%)	5 (6.3%)	1 (5.9%)	0
PFT not done	0	150 (25.6%)	135 (26.2%)	111 (24.3%)	120 (29.1%)	124 (33.7%)	112 (33.9%)	78 (34.8%)	25 (31.6%)	1 (5.9%)	1 (11.1%)

This table shows the frequencies of progression stages. Progression was classified as significant (FVC decline > 10%) or moderate progression (FVC decline 5–10%), stable (FVC decline or increase < 5%) and improvement (FVC increase ≥ 5%). At baseline, 5.5% of patients had a visit before reference and 1.2% no given reference. These patients were excluded from the further analyses. The follow-ups took place every 6 months. FU = Follow-up visit, PFT = pulmonary function test

**Fig. 2:**
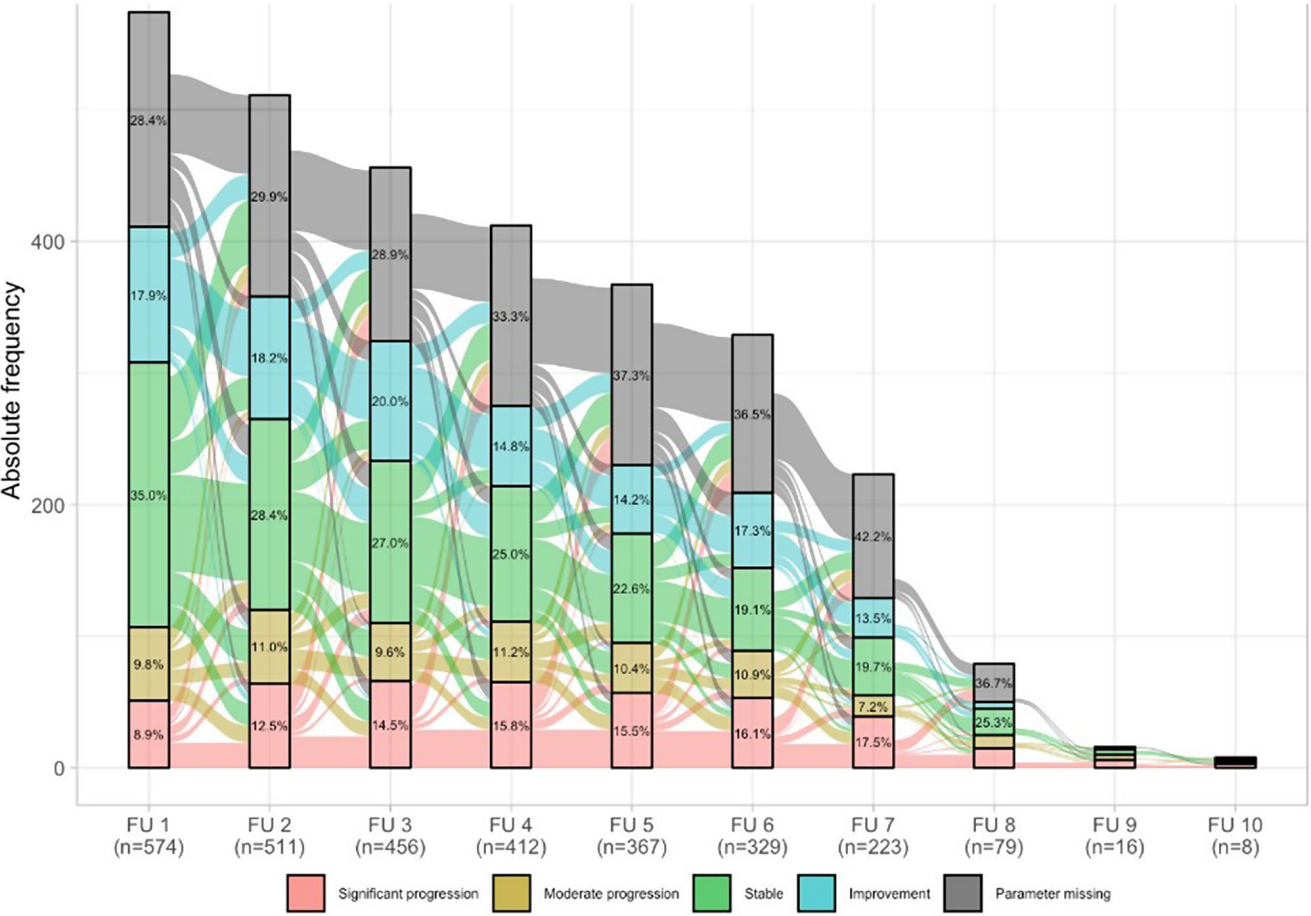
Alluvial plot with transitions of progression stages. This figure shows the transition between different stages of progression. These are presented in different colours for significant progression, moderate progression, stable, improvement or missing parameters. The frequencies are shown for each follow-up visit (FU) starting with the first follow-up visit (FU 1) after 6 months, FU 2 after 12 months, FU 3 after 18 months, etc

Transition between different progression stages is shown in Fig. 2. Mainly, disease trajectory remained similar or progression occurred over time; only a minority of patients improved or had a more severe or less severe deterioration compared to the first follow-up.

### Characterisation of progression

**Fig. 3:**
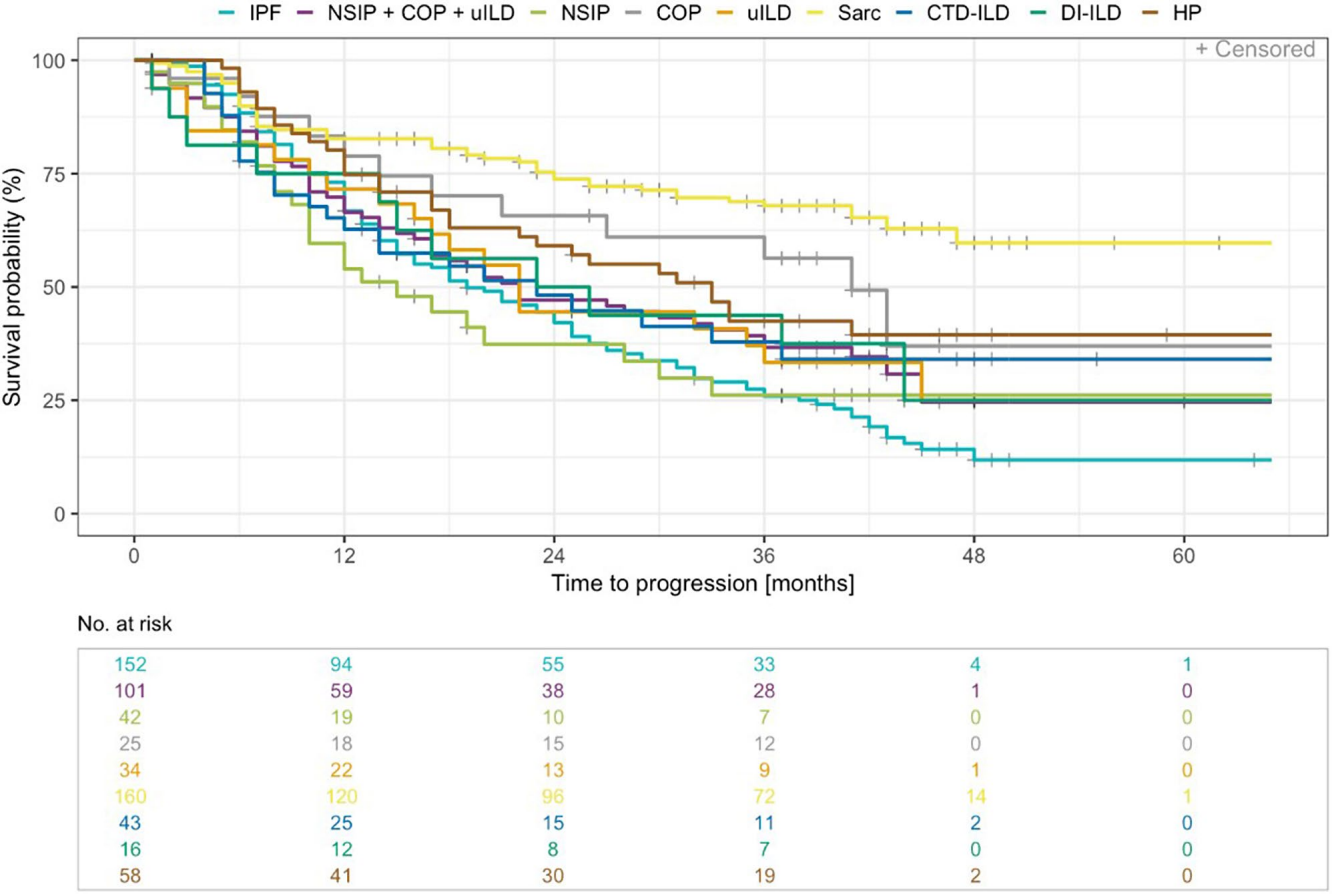
Time to ILD progression in months - Kaplan Meier curve by ILD subtypes of special interest. ILD subtypes of special interest included IPF, iNSIP, COP, uILD, sarcoidosis, HP, CTD-ILD, DI-ILD (n = 530). In addition, iNSIP, COP and uILD were grouped

Median time to ILD progression based on absolute changes in FVC % predicted was 18.9 months in IPF (range 1.1 to 47.54 months) compared to 14.6 months in iNSIP (range 0.9 to 32.69 months) and 32.4 months in HP (range 4.8 to 40.28 months). Significant differences were found between sarcoidosis and IPF (HR = 0.31, p < 0.001), COP and IPF (HR = 0.5, p = 0.021), and HP and IPF (HR = 0.6, p = 0.011; Table 3; Fig. 3).

Regarding the second definition of PF-ILDs, 57.8% met the PFT criterion and 40.6% were hospitalized due to a respiratory cause. In 1.6% both reasons were present. Subsequently, PF-ILD were compared with stable ILD and IPF as reference population. Compared to IPF, patients with PF-ILD were significantly less often male (p < 0.001), younger (p = 0.006), younger at onset of first symptoms (p < 0.001) less often ex-smokers (p = 0.028), and had better baseline values for TLC (p < 0.001). Baseline characteristics between PF-ILD and stable ILD showed no significant differences.

### Risk factors for progression

Univariate logistic regression revealed that baseline value of FVC (OR = 1.02, p = 0.003) as well as age at inclusion (OR = 1.02, p = 0.003) were significant predictors of progression.

In multivariate analyses, significant predictors for progression were reduced baseline FVC together with age (OR = 1.00, p < 0.001). For changes in FVC, significant interaction effects were shown between time since baseline visit and BMI (p = 0.023) and time since baseline visit and the presence of the ILD subtypes IPF (p = 0.001), HP with unknown antigen (p = 0.001), iNSIP (p = 0.025) or CTD-ILD (p = 0.042). The baseline value of FVC was also significantly predictive for changes in FVC, but without a time interaction effect (p = 0.001).

### Survival time

Median survival time since ILD diagnosis was 15.5 years (range 0.1 to 34.4 years), and death was observed in 171 cases (28.5%), mainly due to ILD (n = 71, 41.5%). During the time in the registry, deaths occurred early with 35.1% (n = 60) in the first 12 months and 28.1% (n = 48) in the second year (supplement table S1). Median survival time from inclusion in the registry was 58.7 months [50.1; n.e.].

**Fig. 4:**
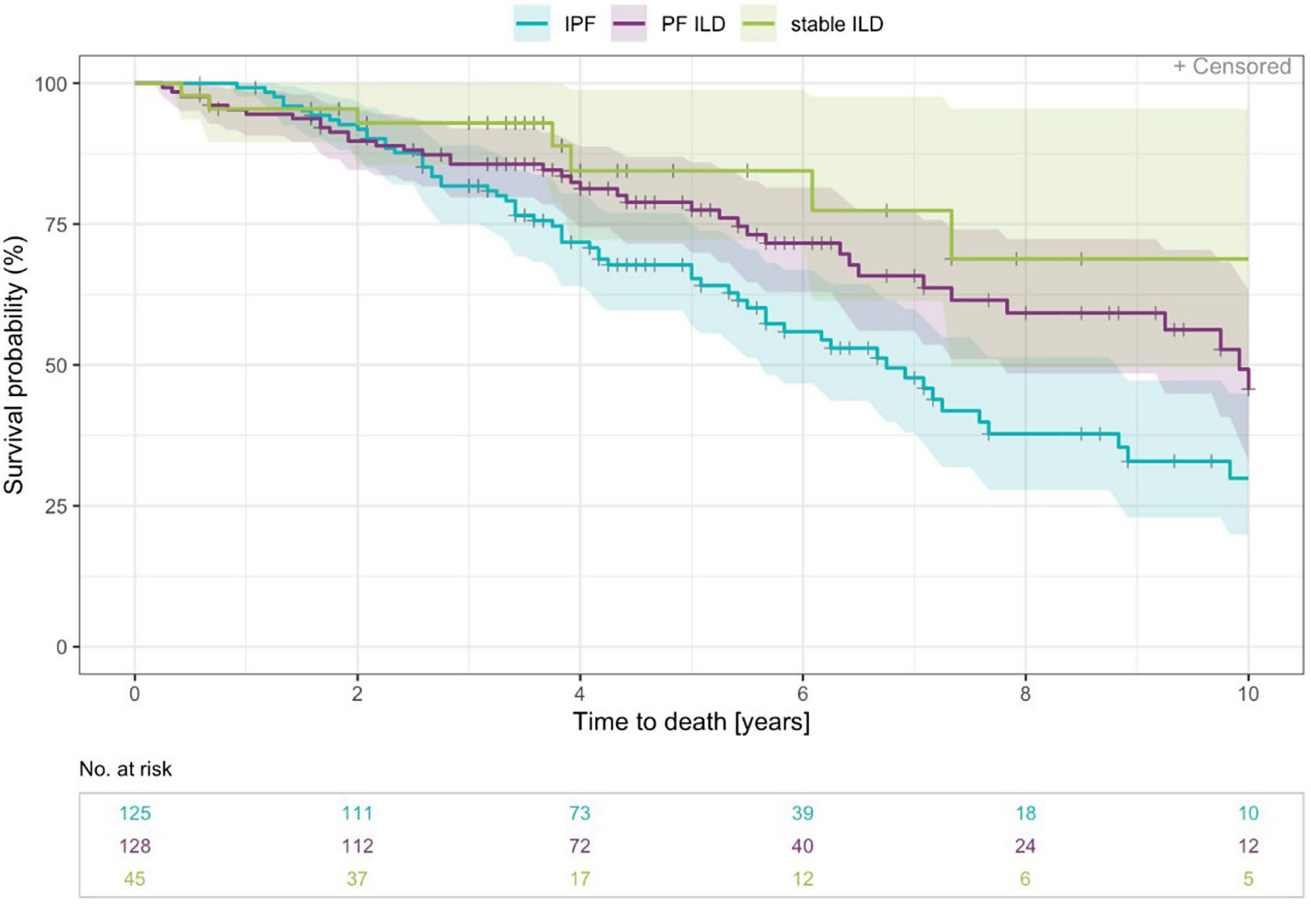
Survival time - Kaplan Meier curve (years 0–10) by IPF, PF-ILD, and stable ILD. The definition for PF-ILD was set as absolute decline in FVC % predicted ≥ 10% within a period of 24 months or at least one respiratory hospitalisation

Median survival time since ILD diagnosis in PF-ILD was 9.9 years (range 0.2 to 22.6). Patients with PF-ILD had a better prognosis than those with IPF (HR = 0.62, p = 0.013; Fig. 4).

### Risk factors for survival time

Higher ILD-GAP indices were a significant risk factor for shorter survival time (GAP stage II vs. I HR = 4.95, p < 0.001; GAP stage III vs. I HR = 9.06, p < 0.001; Table 3). Patients with at least one reported ILD exacerbation (AE) had a median survival time of 7.3 years (range 0.1 to 34.4 years) compared to 19.6 years (range 0.3 to 19.6 years) in patients without exacerbations (HR = 0.39, p < 0.001, Table 3, supplement figure S1). Median survival time after the diagnosis of an AE-ILD was 14.8 months (range 0.1 to 45.6 months). Patients with a long-term oxygen therapy (LTOT) showed worse prognoses with shorter survival time (HR = 0.26, p < 0.001, Table 3).

**Table 3:** Influencing factors on survival time and time to progression

N = 530		Survival time (years)	Survival time vs. IPF		Time to progression (months)	Time to progression vs. IPF	
		Median (range)	HR [95% CI]	p value (Wald test)	Median (range)	HR [95% CI]	p value (Wald test)
ILD Subtype	IPF (n = 152)	5.8 (0.6–14.5)			18.9 (1.1–47.54)		
	iNSIP (n = 42)	9.2 (0.4–18.3)	0.83 [0.47, 1.48]	0.535	14.6 (0.9–32.69)	1.08 [0.7, 1.67]	0.728
	COP (n = 25)	n.e. (0.7–3.2)	0.53 [0.21, 1.31]	0.171	40.1 (1.7–42.02)	0.5 [0.27, 0.9]	0.021
	uILD (n = 34)	5.6 (0.2–5.6)	0.97 [0.54, 1.75]	0.923	21.6 (0.1–44.94)	0.8 [0.5, 1.28]	0.351
	Sarcoidosis (n = 160)	n.e. (1.2–34.4)	0.33 [0.18, 0.63]	< 0.001	n.e. (0.2–46.59)	0.31 [0.22, 0.43]	< 0.001
	HP (n = 58)	16.0 (1.6–22.6)	0.3 [0.17, 0.53]	< 0.001	32.4 (4.8–40.28)	0.6 [0.4, 0.89]	0.011
	CTD-ILD (n = 43)	15.5 (0.6–15.5)	0.33 [0.18, 0.6	< 0.001	22.2 (3.0–36.47)	0.78 [0.5, 1.21]	0.262
	DI-ILD (n = 16)	6.0 (0.2–6.0)	1.41 [0.68, 2.93]	0.358	23.7 (0.5–43.43)	0.75 [0.41, 1.4]	0.372
**N = 601**		**Survival time (years)**					
		**Median (range)**	**HR** **[95% CI]**	**p value** **(Wald test)**			
ILD-GAP Index	II vs. I		4.95 [3.02, 8.11]	< 0.001			
	III vs. I		9.06 [5.58, 14.71]	< 0.001			
	No PFT vs. I		4.34 [2.13, 8.83]	< 0.001			
AE-ILD	AE-ILD (n = 124)	7.3 (0.1–34.4)	0.39 [0.29, 0.53]	< 0.001			
	No AE-ILD (n = 477)	19.6 (0.3–19.6)					
LTOT	LTOT (n = 164)	6.1 (0.1–34.4)	0.26 [0.19, 0.36]	< 0.001			
	No LTOT (n = 437)	n.e. (0.2–19.1)					

Table 3 shows median survival time (in years) and median time to progression (in months) for a selection of ILD subtypes of special interest (n = 530) as well as output of Cox regression model of survival time and time to progression analyses for ILD subtypes compared to IPF. Time to progression was defined according to the definition of progression free survival (PFS; Δ FVC ≥ 10% or Δ DLCO-SB ≥ 15% or death). In addition, output of Cox regression model of survival time was calculated for the full analysis set (FAS, n = 601) by ILD-GAP Index, acute exacerbations (AE), and long-term oxygen therapy (LTOT). AE = acute exacerbations, CI = confidence interval, COP = cryptogenic organizing pneumonia, CTD-ILD = Rheumatic and connective tissue diseases with pulmonary involvement, DI-ILD = drug-related ILD, HP = hypersensitivity pneumonitis HR = Hazard Ratio, iNSIP = non-specific interstitial pneumonia, IPF = idiopathic pulmonary fibrosis, LTOT = long-term oxygen therapy, No PFT = No pulmonary function testing, uILD = unclassifiable ILD.

Outcome analyses by ILD subtypes were performed for a selection of ILD of special interest: IPF, iNSIP, COP, uILD, sarcoidosis, HP, CTD-ILD, DI-ILD. Survival time differed between ILD subtypes (Table 3). For IPF, median survival time was 5.8 years (n = 152, range 0.6 to 14.5 years). The longest median survival time was found in CTD-ILD with 15.5 years (n = 43, range 0.6 to 15.5 years) and the shortest in uILD with 5.6 years (n = 34, range 0.2 to 5.6 years). A significant shorter survival time was found in IPF compared to sarcoidosis (HR = 0.04, p < 0.001), CTD-ILD (HR = 0.33, p < 0.001), and HP (HR = 0.30, p < 0.001, Table 3).

## Discussion

In the present study we report, that within the EXCITING-ILD registry, a registry comprising all ILD entities with prevalent and incident patients, more than half of the patients demonstrated a clinically relevant progression.

Our definition of progression was based on pulmonary function parameters according to the definition used by Hoffmann-Vold et al. for systemic sclerosis-associated ILD (SSc-ILD) [16]. FVC has been used in many studies of fibrotic ILD to characterise disease progression, e.g. in the INBUILD trial [8], another trial on uILD [10], and the RELIEF trial [9]. However, especially in moderate progressions with smaller FVC declines or fluctuating values as shown for SSc-ILD [16], the addition of a further criterion might be reasonable. Furthermore, an international survey emphasised significant delays in the detection of progression in ILD resulting in 25–50% of patients witout adequate therapy [13]. Accordingly, our definition of progression also included hospitalisations known to impact mortality and other outcomes in patients with different ILD [18, 19]. An analysis for hospitalisations based on the EXCITING-ILD registry showed an association of mortality with all cause, ILD-related and respiratory-related hospitalisations for all ILD [15]. It can be assumed that the risk for non-elective hospitalisations is higher in progressive ILD and thus hospitalisation may indicate progression [15, 20].

Frequency of progression within the EXCITING-ILD registry is comparable with other registries, e.g. in the Canadian Registry for fibrosing ILD (CARE-PF), with 50% out of 2746 patients showing a progressive course of disease after two years [11]. Within the CARE-PF registry, progression was defined as FVC decline ≥ 10%, death, lung transplantation or any two of: relative FVC decline ≥ 5% and < 10%, worsening respiratory symptoms or worsening fibrosis on computed tomography of the chest, all within 24 months of diagnosis [11]. Based on the CARE-PF registry and our results also including FVC decline as main parameter for future progression, it can be stated that FVC decline is a reliable indicator of future progression in all ILD. The PROGRESS study based on a cohort of patients with progressive fibrosing ILD found criteria of progression to be fulfilled in 27% of patients over a period of seven years [12]. Progression criteria were defined as a FVC decline ≥ 10%, FVC decline between 5% and 10% associated with worsening of respiratory symptoms or increased extent of fibrosis on chest HRCT, or increased extent of fibrosis on chest HRCT with worsening of respiratory symptoms [12]. Remarkably, in the PROGRESS study, progression was detected less frequently as compared to the CARE-PF and EXCITING-ILD registries. Main reasons for these differences might be the exclusion of patients with IPF in the PROGRESS study in contrast to EXCITING-ILD and CARE-PF registry, as well as differing definitions of ILD progression without additional criteria as hospitalisations, death, or lung transplantations within the PROGRESS study.

The probability of progression increases over time [11] and a transition to more severe stages of disease progression becomes more likely. Also, within the EXCITING-ILD registry, disease trajectory remained similar or progression occurred over time. Only a minority of patients improved or had a more severe or less severe deterioration compared to the first follow-up.

Progression results in worse outcomes and higher mortality. As described by Hambly et al. for progressive fibrosing ILD, IPF showed worse outcomes compared to sarcoidosis and CTD-ILD [11]. Only unclassified ILD had shorter survival times. To complement this, Torrisi et al. reported worse prognosis for unclassifiable ILD and a progressive phenotype [21]. We here report, that mortality was caused to a large extent directly by the ILD. Again, this reflects the high burden of disease in ILD as described by a recent update of the global burden of disease study [22].

The most important predictor for progression within the EXCITING-ILD registry was a reduced baseline FVC, also in conjunction with older age. The CARE-PF registry also found highest risk for progression in patients with reduced PFT [11]. These findings are underlining the value of the ILD-GAP index, a point scoring stage model based on the ILD subtype, gender, age and pulmonary function parameters (FVC and DLCO) to predict mortality in patients with different ILD [17]. Another important risk factor for ILD progression are acute exacerbations, as also reflected in our analyses with a significantly impaired survival after AE-ILD compared to patients not experiencing AE-ILD. The mortality risk after AE is well established for IPF [23], but only sparsely reported in other ILD such as HP [24] and very recently for progressive fibrosing ILD other than IPF [25].

Here, we report a significant better prognosis in HP than in IPF. This is in contrast to the Canadian registry demonstrating similar prevalence for progression in HP and IPF [11]. This discrepancy might be explained by different inclusion criteria as the CARE-PF registry only enrolled patients with fibrosing ILD [11]. The EXCITING-ILD registry included all different ILD regardless of a fibrosing phenotype.

Further strengths of the prospective EXCITING-ILD registry include the reflection of the “real world”-situation of patients with different ILD being treated in different institutions of the health care system including general pulmonology outpatient practice to ILD expert centers. Another advantage is the broad inclusion of all ILD. The large number of patients and the availability of many different parameters allows special questions to be investigated in detail. Our findings highlight the impact of ILD progression on outcome and mortality and are therefore of high value. Our data support the value regular clinical and functional monitoring of ILD patients, especially for those with a risk for future progression [26].

However, some limitations of our approach should be mentioned. Since both incident and prevalent patients were included, no distinctions are possible in this respect. In addition, causal statements cannot be made due to the observational and nonrandomised study character. Furthermore, as baseline CTs are not available, outcomes cannot be distinguished between fibrotic and inflammatory driven ILD. Moreover, not all diagnoses, especially for sarcoidosis, were made on an interdisciplinary basis. Clinical decisions of the physicians may differ, also due to the recruitment from many different centers and therefore levels of expertise in ILD [14]. Another limitation is that comorbidities were not considered thoroughly, although they pose an important role as shown for the prediction of survival in IPF by TORVAN model considering comorbidities in addition to ILD-GAP index [27]. Because the EXCITING-ILD registry was conducted before the establishment of the ATS/ERS/JRS/ALAT Clinical Practice Guidelines on PPF [4], our definition of progression differs. In particular, radiological progression is not considered due to lack of data on CT imaging and clinical symptoms were not reported.

## Conclusion

In the EXCITING-ILD registry, progression was common with more than 50%, and resulted in higher mortality. The most important risk factor was a reduced baseline forced vital capacity. Furthermore, acute exacerbations and respiratory hospitalisations were associated with a significant higher mortality. Early detection of progression remains challenging, especially in patients with only moderate FVC declines. In these cases, further clinical criteria as hospitalisations might be helpful.

### Electronic supplementary material

Below is the link to the electronic supplementary material.

Supplementary Material 1

## Data Availability

The datasets used and/or analysed during the current study are available from the corresponding author on reasonable request.

## Abbreviations

AE: ILD acute exacerbation of ILD
BMI: body mass index
CARE-PF: Canadian Registry for Pulmonary Fibrosis
CI: confidence interval
CO: Pcryptogenic organizing pneumonia
CPFE: Fibrosis in emphysema patients without signs of other ILD
CTD-ILD: connective tissue disease-associated ILD
DI-ILD: drug-related ILD
DIP: desquamative interstitial pneumonia
DLCO-SB: diffusing capacity for carbon monoxide (CO) – single breath
EXCITING-ILD: Exploring Clinical and Epidemiological Characteristics of Interstitial Lung Diseases
FAS: full analysis set
FEV1: forced expiratory volume in 1 s
fILD: fibrotic ILD
FU: follow up
FVC: forced vital capacity
GRAN-ILD: granulomatous ILD
HP: hypersensitivity pneumonitis
HR: Hazard ratios
ILD: interstitial lung disease
iNSIP: non-specific interstitial pneumonia
IPF: idiopathic pulmonary fibrosis
LAM: pulmonary lymphangioleiomyomatosis
LTOT: long-term oxygen therapy
OR: Odd’s ratio
PAP: pulmonary alveolar proteinosis
PFT: pulmonary function testing
PF-ILD: progressive fibrosing ILD
PFS: progression free survival
PLCH: pulmonary Langerhans´ cell histiocytosis
PPF: progressive pulmonary fibrosis
SD: standard deviation
RB-ILD: respiratory bronchiolitis-associated interstitial lung disease
RTX-ILD: radiotherapy associated ILD
SSc: systemic sclerosis
SD: standard deviations
uILD: unclassifiable ILD
VC: vital capacity
6-MWD: 6 min walking distance
