# Supplementary material for: Disease trajectories in interstitial lung diseases – data from the EXCITING-ILD registry
Source: Respir Res. 2024 Mar 6;25:113. doi: 10.1186/s12931-024-02731-3 (PMC10919020; doi:10.1186/s12931-024-02731-3)
Supplement: Supplementary file 1 — Supplementary Material 1 [file 12931_2024_2731_MOESM1_ESM.docx]

**Supplement**

|  | | | | | | **Number of events in:** | | | | |
| --- | --- | --- | --- | --- | --- | --- | --- | --- | --- | --- |
| **N** | **Events** | **Censored** | **Median survival time in months**  **[95% CI]** | **Min** | **Max** | **Months 1-12** | **Months 13-24** | **Months 25-36** | **Months**  **37-48** | **Months 49-60** |
| 601 | 171 (28.5%) | 430 (71.5%) | 58.7 [50.1, n.e.] | 0.1 | 58.7 | 60 (35.1%) | 48 (28.1%) | 35 (20.5%) | 25 (14.6%) | 3  (1.8%) |

**Table S1 Summary of survival time in months since date of inclusion analysis.**

Survival time was defined as time between date of death and date of inclusion + 1 day in months. Individuals who do not experience death until the registry was closed, who were lost to follow up or withdraw from the registry are censored. N= number of patients at risk, CI= confidence interval.


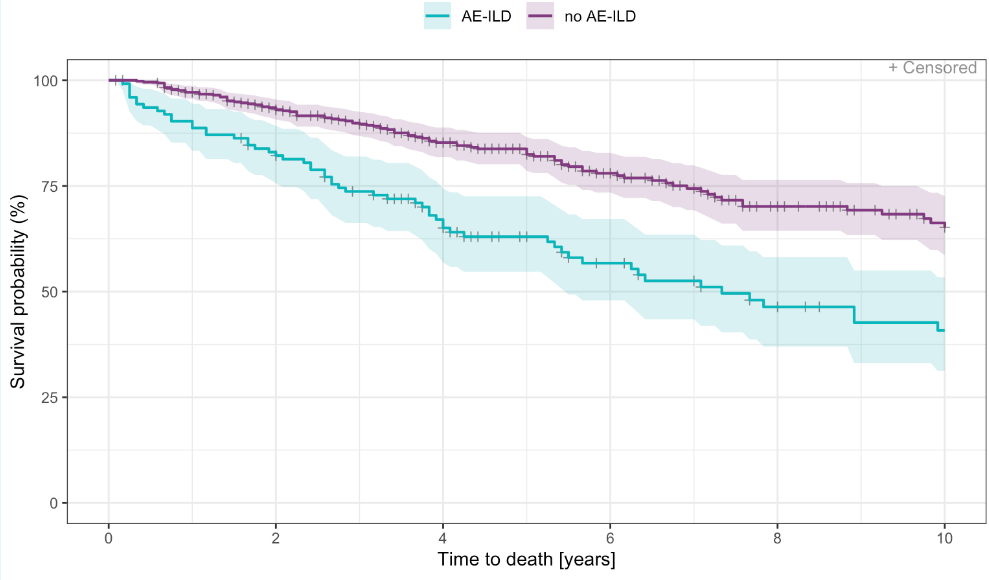


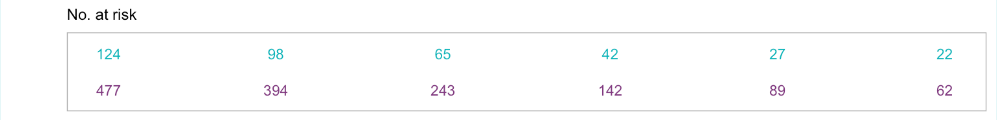


**Figure S1 Survival time in years - Kaplan Meier curve (years 0-10) by AE-ILD/no AE-ILD**

Survival time was defined as time between date of death and date of inclusion + 1 day. All patients with a recorded acute exacerbation were counted as AE-ILD.
